# Evaluation of hirst-type spore trap to monitor environmental fungal load in hospital

**DOI:** 10.1371/journal.pone.0177263

**Published:** 2017-05-09

**Authors:** Cédric Dananché, Marie-Paule Gustin, Pierre Cassier, Sophie Tiphaine Loeffert, Michel Thibaudon, Thomas Bénet, Philippe Vanhems

**Affiliations:** 1 Unité d’hygiène, épidémiologie et prévention, Groupement Hospitalier Centre, Hospices Civils de Lyon, France; 2 Laboratoire des Pathogènes Emergents—Fondation Mérieux, Centre International de Recherche en Infectiologie (CIRI), Inserm U1111, CNRS UMR5308, ENS de Lyon, France; 3 Département de santé publique, Institut des Sciences Pharmaceutiques et Biologiques (ISPB)–Faculté de Pharmacie, Université Claude Bernard Lyon 1, France; 4 Laboratoire de Biologie Sécurité Environnement, Groupement Hospitalier Centre, Hospices Civils de Lyon, France; 5 European Aerobiology Society and Réseau National de Surveillance Aérobiologique, Brussieu, France; Ecole des Mines d'Ales, FRANCE

## Abstract

The main purpose was to validate the use of outdoor-indoor volumetric impaction sampler with Hirst-type spore traps (HTSTs) to continuously monitor fungal load in order to prevent invasive fungal infections during major structural work in hospital settings. For 4 weeks, outdoor fungal loads were quantified continuously by 3 HTSTs. Indoor air was sampled by both HTST and viable impaction sampler. Results were expressed as particles/m^3^ (HTST) or colony-forming units (CFU)/m^3^ (biocollector). Paired comparisons by day were made with Wilcoxon’s paired signed-rank test or paired Student’s t-test as appropriate. Paired airborne spore levels were correlated 2 by 2, after log-transformation with Pearson’s cross-correlation. Concordance was calculated with kappa coefficient (κ). Median total fungal loads (TFLs) sampled by the 3 outdoor HTSTs were 3,025.0, 3,287.5 and 3,625.0 particles/m^3^ (*P* = 0.6, 0.6 and 0.3).—Concordance between *Aspergillaceae* fungal loads (AFLs, including *Aspergillus* spp. + *Penicillium* spp.) was low (κ = 0.2). A low positive correlation was found between TFLs sampled with outdoor HTST and indoor HTST with applying a 4-hour time lag, r = 0.30, 95% CI (0.23–0.43), *P*<0.001. In indoor air, *Aspergillus* spp. were detected by the viable impaction sampler on 63.1% of the samples, whereas AFLs were found by HTST-I on only 3.6% of the samples. Concordance between *Aspergillus* spp. loads and AFLs sampled with the 2 methods was very low (κ = 0.1). This study showed a 4-hour time lag between increase of outdoor and indoor TFLs, possibly due to insulation and aeraulic flow of the building. Outdoor HTSTs may permit to quickly identify (after 48 hours) time periods with high outdoor fungal loads. An identified drawback is that a too low sample area read did not seem to enable detection of *Aspergillaceae* spores efficiently. Indoor HTSTs may not be recommended at this time, and outdoor HTSTs need further study. Air sampling by viable impaction sampler remains the reference tool for quantifying fungal contamination of indoor air in hospitals.

## Introduction

Environmental fungal contamination by moulds, such as *Aspergillus* spp., during major hospital renovation is a serious risk factor for outbreaks of nosocomial invasive fungal infections (IFI) in immunocompromised patients. During periods of demolition, unprotected indoor air spore concentrations in nearby buildings increase noticeably [1], and outdoor levels of thermotolerant fungi could rise 10^5^-fold compared to pre-demolition values [2]. Air sampling is an interesting tool to check efficiency of barrier measures in order to prevent IFI in high-risk patients [3]. The reference tool for monitoring fungal loads in hospitals is currently the viable impaction sampler (biocollector) [4,5]. However, air sampling is time-consuming, and results are obtained several days after sampling, as fungal colonies needs time to grow. If fungal load in air is increasing, the same time interval is needed to detect the increase and to implement adequate barrier measures for patients; in the meantime, patients have a higher risk to develop IFI. Therefore, there is a need to develop new tools that provide real-time results of air fungal load.

Hirst-type spore traps (HTST) is a volumetric impaction sampler which permit to have results of the air fungal spore load quickly. With this device, both viable and nonviable spores are impacted on a silicon strip, then microscopically read, but the spores are not cultured. Thus, this device may have an interest to monitor fungal loads in air as no growing time is needed. To our knowledge, HTST has never been evaluated for monitoring fungal contamination in hospital setting.

In our university teaching hospital, a large renovation program began in mid-2015 with the demolition of a major central block. Before the work started, we planned to evaluate new methods for continuous monitoring of indoor and outdoor air contamination, in order to calibrate future surveillance. Our objective was to assess the use of outdoor-indoor volumetric methods of air sampling by impaction with 7-day Hirst-type spore traps (HTST) [6,7], Two points were evaluated: (i) the correlation and concordance between fungal loads sampled with several outdoor HTSTs and between fungal loads sampled with outdoor and indoor HTSTs; (ii) the correlation and concordance between indoor fungal loads sampled with HTST and with a viable impaction sampler.

## Materials and methods

### Setting

This pilot study was performed over a 4-week period, between October 23 and November 21, 2013 at Edouard Herriot Hospital, an 850-bed university-affiliated hospital in Lyon, Rhône-Alpes, France. The hospital consists of 32 buildings, with 20 designated for patient care. A large renovation program, consisting of central block demolition, land excavation, foundation work and construction of a new building started in mid-2015 and should last at least 3 years. The hospital has a solid organ transplant program and 4 intensive care units in the vicinity of the working site. The solid transplant unit has a controlled mechanical ventilation system but no additional positive pressure or filtered air.

### Outdoor air samples

**Fig 1:**
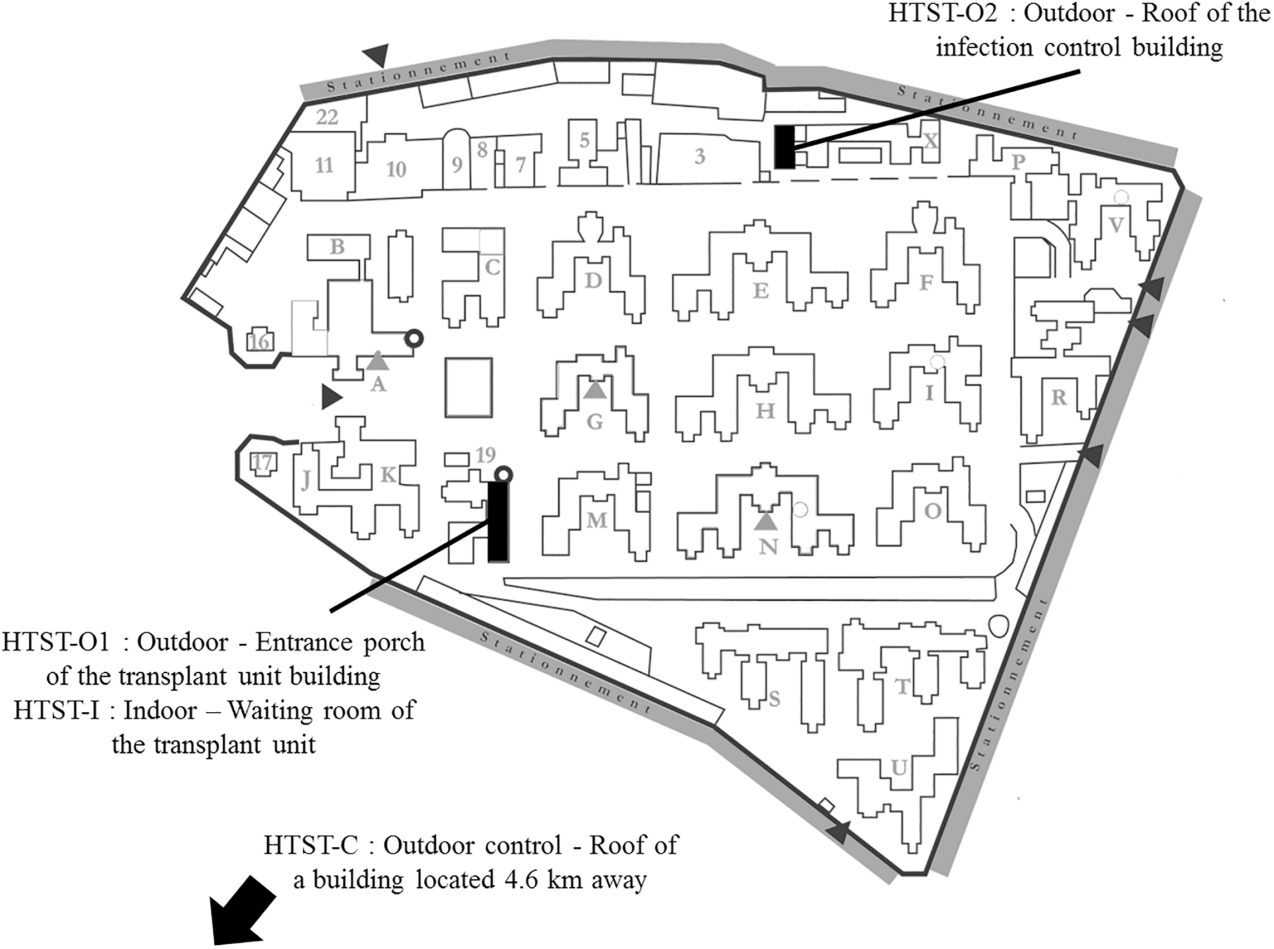
Locations of Hirst-type spore traps (HTST) in the setting.

External air was monitored continuously, by 3 HTSTs (Lanzoni VPPS 2000, Bologna, Italy) placed above the entrance porch of the transplant unit building (HTST-O1, GPS coordinates: 45°44'34.906''N,4°52'52.925''E) and on the roof of the infection control building (HTST-O2, GPS coordinates: 45°44'42.076'' N,4°52'57.715''E, altitude 185m) (approximately 3 and 20 meters high, respectively). A control HTST (HTST-C) was situated outdoors, on the roof of a building outside the hospital, 4.6 km away, 25 meters high in a residential area (GPS coordinates: 45°43'39.90"N, 4°49'29.48"E, altitude 173m). This control HTST permitted to control if correlations were observed between fungal loads sampled with HTSTs located in the hospital compound and fungal loads sampled with HTST located few kilometers away and used for the national surveillance. Locations of HTSTs are described in Fig 1. Particles were impacted at a flow rate of 10 L/min on a silicone strip placed on a drum. Sampler drums were changed weekly for 4 weeks (n = 28 days for HTST-O1 and O2 and n = 29 days for HTST-C). Cellulose strips coated with silicon were divided into 7 segments representing the impact of the previous 7 days. Each segment was read at 2-hour intervals by optical microscope (Axiostar, Carl Zeiss, Göttingen, Germany) with 400x magnification. With this device, all spores are counted, and thereby the differentiation between viable and nonviable spores is not possible. Total fungal loads (TFLs) and *Aspergillaceae* fungal loads (AFLs) (including *Aspergillus* spp. + *Penicillium* spp.) were quantified. TFLs might be interpreted as a reflection of the global fungal load in the air. We specifically focused *Aspergillaceae* fungal loads because of their high involvement in human infections. Of note, this method did not permit to distinguish *Aspergillus* spp. from *Penicillium* spp. According to the technical specification CEN/TS 16868 [8], fungal spore counts should be expressed as the daily average fungal spores counts per cubic meter of air (particles/m^3^). For this purpose, the number of fungal spores counted is multiplied by a conversion factor that takes into account the volume of air sampled, the sampling area and the size of the microscope’s field of view used. Formulae used to calculate spore concentrations, conversion factor and proportions of surface read were as follows:

$$\%\;of\;surface\;read=\frac{S\;total\;sampled}{S\;analysed}\times 100$$

$$CF=\frac{S\;total\;sampled}{S\;analysed}\times \frac{1}{V}$$

Pollenorfungalsporeconcentration=n×CF

where S: surface (total sampled or analysed) in mm^2^, V: volume of sucked air in m^3^, n: number of fungal spores counted in the analysed area of the microscope slide. The conversion factor used was 25.7 (ie. 0.27% of the surface sampled was read).

### Indoor air samples

Indoor air was monitored continuously by HTST placed in the waiting room of the transplant unit (HTST-I, unprotected internal air), at 80 cm high, with the same settings as the outdoor HTSTs (n = 28 days) (Fig 1). The first conversion factor used was 25.7 (ie. 0.27% of the surface sampled was read). An extended area of some samples was read as a second step, with a conversion factor of 0.19 (ie. 36.5% of the surface sampled was read). It was also monitored with a viable impaction air sampler device known as a biocollector (AirIDEAL, BioMérieux, Marcy l’Étoile, France), in the same waiting room, at 1 meter high. Paired indoor air samples were collected at the same place twice daily (at 10 a.m. and 2 p.m. ± 2h), from Monday to Friday, excluding public holidays (n = 19 days). The biocollector draws in air at 100 L/min for 1 min. Overall, 100 L were collected on Sabouraud Dextrose Chloramphenicol agar (BioMérieux) and incubated for 5–7 days at 30°C for the 1^st^ plate and for 48 hours at 37°C for the 2^nd^ plate. Viable fungal colonies (colony forming units) were counted and identified. TFLs and *Aspergillus* spp. loads were expressed as colony-forming units (CFU)/m^3^.

### Statistical analysis

We expressed continuous variables as medians and interquartile range (IQR) or means ± standard deviation, as appropriate. Paired comparisons by day were made with Wilcoxon’s paired signed-rank test for medians and paired Student’s t-test for means. Medians also underwent multiple comparisons according to Hochberg’s procedure [9]. Categorical variables were reported as number and percentage; concordance was calculated with kappa coefficient (κ). AFLs and *Aspergillus* spp. loads were categorized as: ≤100, 100–200, ≥200 CFU/m^3^ or particles/m^3^. Paired airborne spore levels sampled every 2 hours were correlated 2 by 2, after log-transformation with Pearson’s cross-correlation. Correlations were also tested with a 2-hour time lag. *P*<0.05 was considered to indicate statistical significance. Analyses were conducted in R language, version 3.0.2.

## Results

### Correlation and concordance between fungal loads sampled with several outdoor HTSTs and between fungal loads sampled with outdoor and indoor HTSTs

**Fig 2:**
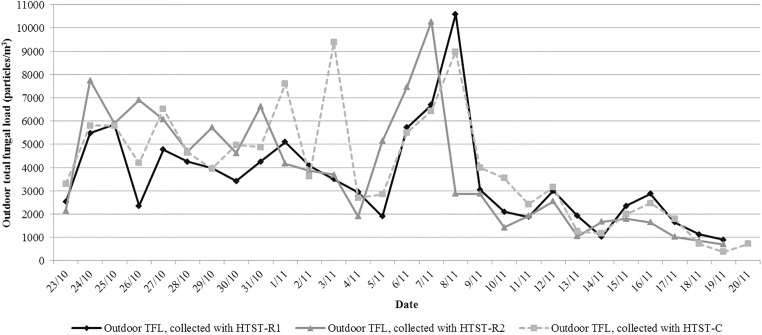
Description of outdoor total fungal loads loads sampled by HTST-O1, -O2 and–C. NOTE: HTST: Hirst-type spore trap. O1 and O2: placed outdoor, above the entrance porch of the transplant unit building (HTST-O1) and on the roof of the infection control building (HTST-O2). C: control, placed outdoor, outside the hospital, 5 km away, in a residential area. The differences between the median total fungal load between HTST-O1, -O2 and -C were not statistically significant (HTST-O1 *vs*. O2: *P* = 0.6, O2 *vs*. C: *P* = 0.6, O1 *vs*. C: *P* = 0.3, with Wilcoxon’s paired signed-rank test using Hochberg’s procedure).

Outdoor and indoor TFLs, AFLs and *Aspergillus* spp. loads are reported in Table 1. Median TFLs sampled by the 3 outdoor HTSTs were 3,025.0 particles/m^3^ (2,056.3–4,381.3), 3,287.5 particles/m^3^ (1,768.8–5,768.8) and 3,625.0 particles/m^3^ (2,425.0–5,500.0) for HTST-O1, O2 and C, respectively (Fig 2). The differences were not statistically significant (HTST-O1 *vs*. O2: W = 169.5 and *P* = 0.6, O2 *vs*. C: W = 190.5 and *P* = 0.6, O1 *vs*. C: W = 129 and *P* = 0.3). TFLs correlated with the 2 outdoor HTSTs located in the hospital compound (HTST-O1 *vs*. O2, r = 0.48, 95% confidence interval [95% CI]: 0.39–0.57, *P*<0.001). The best correlation was obtained without applying any time lag. AFLs were measured on 25.0%, 21.4% and 48.3% of readed samples of HTST-O1, O2 and C, respectively. Concordance was low between HTST-O1 and O2 (Table 2, κ = 0.2). No differences between AFLs medians were discerned during the days when they were ascertained by either HTST-O1 or O2 (n = 11 days): 100.0 particles/m^3^ (0.0–125.0) and 100.0 particles/m^3^ (0.0–162.5), respectively, W = 28.5 and *P* = 0.9. A low positive correlation was found between TFLs sampled with outdoor HTST-O1 and indoor HTST-I with applying a 4-hour time lag, r = 0.30, 95% CI (0.23–0.43), *P*<0.001.

**Table 1:** Description of environmental fungal loads collected by HTSTs and viable impaction sampler.

	Outdoor air collected by HTST-O1 n = 28 days	Outdoor air collected by HTST-O2 n = 28 days	Outdoor air collected by HTST-C n = 30 days	Indoor air collected by HTST-I n = 28 days	Indoor air collected by viable impaction sampler n = 19 days	Wilcoxon statistic (P-value)^c^		
						HTST-O1 *versus* HTST-O2	HTST-O2 *versus* HTST-I	HTST-I *versus* viable impaction sampler
Median TFL^a^, (IQR)^b^	3,025.0 (2,056.3–4,381.3)	3,287.5 (1,768.8–5,768.8)	3,625.0 (2,425.0–5,500.0)	850.0 (687.5–1,162.5)	100.0 (37.5–155.0)	169.5 (0.6)	6.5 (<0.01)	171 (<0.01)
Median AFL or *Aspergillus* spp. load^a^, (IQR)^b^	100.0 (0.0–125.0)	100.0 (0.0–162.5)	125.0 (0.0–200.0)	0.0 (0.0–0.0)	5.0 (0.0–10.0)	28.5 (0.9)	1 (0.05)	12 (0.01)

NOTE: AFL: Aspergillaceae fungal load, HTST: Hirst-type spore trap, IQR: Interquartile range. TFL: Total fungal load.

^a^Indoor air collected by HTSTs was expressed in AFLs, and indoor air collected by viable impaction sampler was expressed in Aspergillus spp. load.

^b^Expressed in particles/m^3^ for HTSTs with conversion factor 25.7 and in CFU/m^3^ for viable impaction sampler.

^c^Wilcoxon statistics and P-values calculated with Wilcoxon’s paired signed-rank test.

**Table 2:** Description of the number of day in which each category of AFL or *Aspergillus* spp. load (0; 1–100; 100–200; >200) were observed ^a^.

	Outdoor air collected by HTST-O1 n = 28 days	Outdoor air collected by HTST-O2 n = 28 days	Outdoor air collected by HTST-C n = 30 days	Indoor air collected by HTST-I n = 28 days	Indoor air collected by viable impaction sampler n = 19 days	κ coefficient (P-value)^b^		
						HTST-O1 *versus* HTST-O2	HTST-O2 *versus* HTST-I	HTST-I *versus* viable impaction sampler
*0*	21 (75.0%)	22 (78.6%)	15 (51.7%)	27 (94.4%)	7 (36.8%)	0.2 (0.1)	0.1 (0.2)	0.1 (0.2)
*1–99*	2 (7.1%)	1 (3.6%)	4 (13.8%)	1 (3.6%)	12 (63.2%)			
*100–199*	4 (14.3%)	3 (10.7%)	6 (20.7%)	0 (0.0%)	0 (0.0%)			
*>200*	1 (3.6%)	2 (7.1%)	4 (13.8%)	0 (0.0%)	0 (0.0%)			

NOTE: AFL: Aspergillaceae fungal load, HTST: Hirst-type spore trap, IQR: Interquartile range. TFL: Total fungal load.

^a^Indoor air collected by HTSTs was expressed in AFLs, and indoor air collected by viable impaction sampler was expressed in Aspergillus spp. load. Data are Expressed in particles/m^3^ for HTSTs with conversion factor 25.7 and in CFU/m^3^ for viable impaction sampler.

^b^κ coefficients and P-values calculated with Kappa test.

### Correlation and concordance between indoor fungal loads sampled with HTST and with a viable impaction sampler

**Fig 3:**
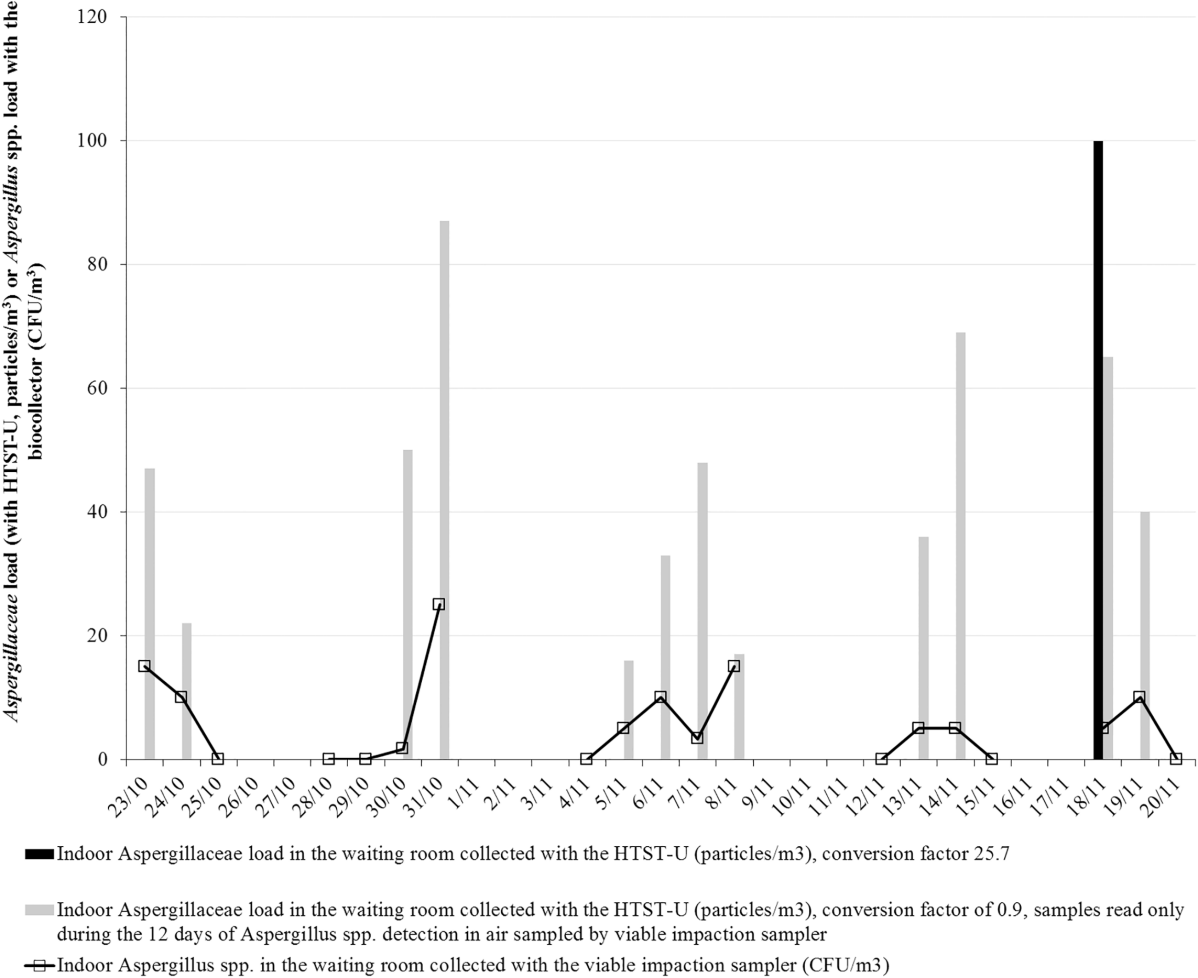
Description of indoor *Aspergillaceae* and *Aspergillus* spp. loads sampled by HTST-I and viable impaction sampler. NOTE: HTST: Hirst-type spore trap. I: placed indoor, in the waiting room of the transplant unit. CFU: Colony-forming units. Air sampled by viable impaction sampler retrieved *Aspergillus* spp. on 12 of 19 days (middle-grey line). *Aspergillaceae* were detected on only 1 of 28 days by HTST-I with conversion factor 25.7 (black line). New reading of the same samples with improved conversion factor (0.19) only on days when *Aspergillus* spp. were retrieved by the viable impaction sampler: it captured *Aspergillaceae* on 12 of 12 days (light-grey line).

TFL and *Aspergillus* spp. load collected with the viable impaction sampler are disclosed in S1 Table. Median indoor TFLs collected by HTST-I were significantly higher than those sampled by biocollector: 850.0 particles/m^3^ (687.5–1,162.5) and 100.0 CFU/m^3^ (37.5–155.0), respectively (W = 171 and *P*<0.01) with Pearson’s correlation coefficient of 0.51 (95% CI: 0.055–0.79, *P* = 0.03). *Aspergillus* spp. were detected by the biocollector on 12 of 19 days (63.1%), whereas AFLs were found by HTST-I on only 1 of 28 days (3.6%) (Fig 3). During the 12 days of *Aspergillus* spp. detection, median indoor *Aspergillus* spp. load collected by the biocollector was 5.0 CFU/m^3^ (0.0–10.0), whereas AFL median measured by HTST-I was zero (W = 12 and *P* = 0.01). Concordance was very low between HTST-I and the biocollector (Table 2, κ<0.1 and *P* = 0.1). While the biocollector was considered to be the gold standard for detecting *Aspergillus* spp., HTST-I sensitivity in identifying *Aspergillaceae* was 5.7%, with a conversion factor of- of 25.7. During the same 12 days of *Aspergillus* spp. detection in air sampled by biocollector, an higher area of the samples were read (conversion factor: 0.19); AFLs were detected on 12 of 12 days (100%) (Fig 3). Mean *Aspergillus* spp. load was 8.4±4.1 particles/m^3^/day for AFLs collected by HTST-I and 9.6±6.8 CFU/m^3^ for *Aspergillus* spp. load collected by biocollector (*P* = 0.6). No significant correlation between-the 2 methods were observed (r = 0.23, 95% CI: [-0.39–0.71], *P* = 0.5). *Aspergillus* species found in air samples collected with the biocollector were *A*. *fumigatus* (9/19 days, 47.4%), *A*. *versicolor* (4/19 days, 21.1%), *A*. *glaucus* (9/19 days, 47.4%), *A*. *candidus* (2/19 days, 10.5%) and *A*. *flavus* (1/19 days, 5.3%).

## Discussion

This pilot study showed low sensitivity of HTSTs in detecting *Aspergillus* spp. spores in indoor air, with a conversion factor of 25.7. It failed to demonstrate correlations between indoor AFLs sampled by HTSTs and *Aspergillus* spp. loads sampled by biocollector. However, it disclosed correlations between outdoor TFLs and AFLs sampled by HTSTs at different outdoor places, and correlation between outdoor and indoor TFLs with applying a 4-hour time lag. Then, an increase of fungal loads sampled outdoor might involve an indoor increase few hours later. This time lag may be due to insulation, aeraulic flow of the building and meteorological and human factors (wind, opening the windows…). Using a real-time method to detect increases of fungal loads in outdoor air may permit to implement adequate barrier measures for patients before increases of indoor fungal loads. Of note, *Aspergillus* spp. were frequently retrieved in unprotected internal air of the transplantation unit (63.1% of the days), with a potential risk for immunocompromised patients.

It is known that indoor fungi can serve as bioindicators of air quality in order to assess fungal risk [10]. Monitoring of outdoor and indoor fungal contamination during major demolition-construction work might help to detect increased fungal loads [11]. Some experts suggest that a shift towards devices providing continuous monitoring of fungal air load may avoid missing spore bursts [12,13]. With this aim, we tested HTSTs as a tool for continuous monitoring of fungal contamination. Outdoor HTSTs may permit us to quickly identify (after 48 hours) time periods with high outdoor fungal loads if their locations are carefully determined: they should be situated close to the work site with respect to the prevailing wind direction. A study lead on outdoor samples showed that viable methods provide underestimates in relation to the non-viable methods [14].

In our study, HTSTs seems to be unsuitable for indoor use, probably because of the lack of air movement, which induces too low airflow on cellulose strip coated with silicon. Moreover, the literature reveals that small spores, such as those of *Aspergillus* and *Penicillium*, were reported to be underestimated, and differentiation between the 2 genera was not possible, as well as between viable and nonviable particles [14–16].

Another identified drawback of HTSTs use is the high sample area that it is necessary to read: indeed, a too low sample area read did not seem to enable detection of *Aspergillaceae* spores efficiently and may underestimates the results. In addition, while sampling is continuous, results are not continuous and immediate because reading by optical microscope is needed.

The main strengths of our study lie in the comparison of 2 methods assessing true fungal contamination, and the availability of a control group. The main limitation was the limited sample size to demonstrate relationships between fungal airway contamination.

To conclude, we determined that indoor HTSTs are not routinely recommended for monitoring of indoor fungal contamination. Air sampling by biocollector remains the reference tool for quantifying fungal contamination of indoor air during hospital demolition-construction work. HTSTs for environmental monitoring of outdoor air need further study. For that purpose, a large-scale investigation, carried out over a 2-year period of hospital demolition and construction, is in progress in our setting.

## Supporting information

S1 Table TFL and *Aspergillus* spp. load collected with the viable impaction sampler and meteorological parameters during the study. (XLSX)
